# Neurocognitive test performance following cancer among middle‐aged and older adults in the Hispanic Community Health Study/Study of Latinos (HCHS/SOL) and the SOL‐Investigation of Neurocognitive Aging Ancillary Study

**DOI:** 10.1002/cam4.5863

**Published:** 2023-03-31

**Authors:** Humberto Parada, Margaret S. Pichardo, Linda C. Gallo, Gregory A. Talavera, Corinne McDaniels‐Davidson, Frank J. Penedo, David J. Lee, Wassim Tarraf, Tayna P. Garcia, Martha L. Daviglus, Hector M. González

**Affiliations:** ^1^ Division of Epidemiology and Biostatistics, School of Public Health San Diego State University San Diego California USA; ^2^ UC San Diego Health Moores Cancer Center La Jolla California USA; ^3^ Department of Surgery Hospitals of the University of Pennsylvania Philadelphia Pennsylvania USA; ^4^ Department of Psychology San Diego State University San Diego California USA; ^5^ Division of Health Promotion and Behavioral Science, School of Public Health San Diego State University San Diego California USA; ^6^ Departments of Psychology and Medicine University of Miami College of Arts and Sciences and Miller School of Medicine Miami Florida USA; ^7^ Department of Public Health Sciences University of Miami Miller School of Medicine Miami Florida USA; ^8^ Institute of Gerontology and Department of Healthcare Sciences Wayne State University Detroit Michigan USA; ^9^ Department of Biostatistics University of North Carolina at Chapel Hill Chapel Hill North Carolina USA; ^10^ Institute for Minority Health Research University of Illinois Chicago Chicago Illinois USA; ^11^ Department of Neurosciences University of California, San Diego La Jolla California USA

**Keywords:** cancer, cancer survivors, cognitive decline, cognitive function, neurocognitive testing

## Abstract

**Background:**

Cancer patients and survivors often experience acute cognitive impairments; however, the long‐term cognitive impact remains unclear particularly among Hispanics/Latinos. We examined the association between cancer history and neurocognitive test performance among middle‐aged and older Hispanic/Latinos.

**Methods:**

Participants included 9639 Hispanic/Latino adults from the community‐based and prospective Hispanic Community Health Study/Study of Latinos. At baseline (2008–2011; V1), participants self‐reported their cancer history. At V1 and again at a 7‐year follow‐up (2015–2018; V2), trained technicians administered neurocognitive tests including the Brief‐Spanish English Verbal Learning Test (B‐SEVLT), Word Fluency Test (WF), and Digit Symbol Substitution Test (DSS). We used survey linear regression to estimate the overall, sex‐specific, and cancer site‐specific [i.e., cervix, breast, uterus, and prostate] adjusted associations between cancer history and neurocognitive test performance at V1 and changes from V1 to V2.

**Results:**

At V1, a history of cancer (6.4%) versus no history of cancer (93.6%) was associated with higher WF scores (β = 0.14, SE = 0.06; *p* = 0.03) and global cognition (β = 0.09, SE = 0.04; *p* = 0.04). Among women, a history of cervical cancer predicted decreases in SEVLT‐Recall scores (β = −0.31, SE = 0.13; *p* = 0.02) from V1 to V2, and among men, a history of prostate cancer was associated with higher V1 WF scores (β = 0.29, SE = 0.12; *p* = 0.02) and predicted increases in SEVLT‐Sum (β = 0.46, SE = 0.22; *p* = 0.04) from V1 to V2.

**Conclusion:**

Among women, a history of cervical cancer was associated with 7‐year memory decline, which may reflect the impacts of systemic cancer therapies. Among men, however, a history of prostate cancer was associated with improvements in cognitive performance, perhaps due in part to engaging in health promoting behaviors following cancer.

## Graphical abstract

We examined the associations between a history of cancer and neurocognitive test performance cross‐sectionally and longitudinally over a 7‐year follow‐up among diverse middle‐aged and older Hispanic/Latino adults enrolled in the Hispanic Community Health Study/Study of Latinos (HCHS/SOL). Here, we report that a history of cervical cancer was associated with 7‐year declines in memory among cognitively healthy, diverse, and middle‐aged Hispanic/Latina women, and with better performance on tests of language and executive functioning and episodic learning and verbal memory and with improved overall global cognition over time among men. This work is critical to understanding the impact of cancer on cognition among US Hispanics/Latinos, particularly as this population ages.

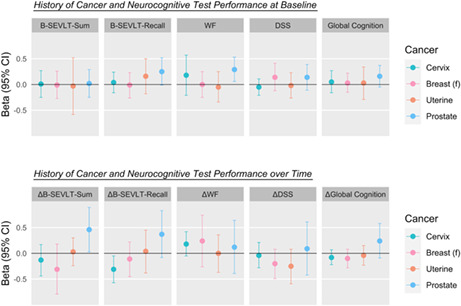

## 1 INTRODUCTION

Cancer‐related cognitive impairment (CRCI) is reported up to 75% of cancer patients during treatment.^1^ Furthermore, up to 35% of cancer survivors continue to experience CRCI years after treatment.^1^ Most cognitive domains appear to be affected including attention, memory, executive function, and multitasking.^2^ The pleiotropic effects on cognition are not surprising given the physical (e.g., fatigue^3^ and hearing loss^4^), psychological (e.g., anxiety and depression, and emotional distress^5^), and biological (e.g., DNA damage^6^ and telomere shortening^7^) impacts of cancer or its treatments. Although the mechanisms remain to be fully elucidated, in addition to direct neurotoxic injuries, cancer treatments are hypothesized to impact cognition as a result of increases in oxidative stress and neuroinflammation, accelerated aging processes, and changes in hormones important for normal cognitive function.^8^ Cancer may also affect cognitive reserve or initiate dementia pathology thus potentially impacting long‐term trajectories of cognitive function. As more cancer patients including Hispanics/Latinos survive into older adulthood,^9^ understanding the long‐term impacts of cancer on cognition and in particular the impacts of cancer at midlife on cognition in late‐life is essential to developing intervention strategies to mitigate these risks.

Epidemiologic studies of cognitive function following cancer conducted to date have primarily focused on affluent, highly educated, non‐Hispanic White populations,^2^ and have reported increases in acute cognitive impairments following cancer.^10^, ^11^, ^12^, ^13^ Studies of Hispanics/Latinos are limited to just one cross‐sectional study that examined correlates of cognitive functioning among Hispanic/Latina breast cancer survivors.^14^ However, the effects of cancer therapies on cognition may be of particular relevance for Hispanics/Latinos who are the second largest racial or ethnic group in the United States accounting for 19% of the total population,^15^ and who are often diagnosed with cancer at younger ages^16^ and at later stages requiring more aggressive and systemic treatments compared to non‐Hispanic Whites.^17^

To address these knowledge gaps, this study examined the associations between a history of cancer with neurocognitive test performance and with a 7‐year change in neurocognitive test performance among diverse middle‐aged and older Hispanic/Latino adults enrolled in the Hispanic Community Health Study/Study of Latinos (HCHS/SOL). We examined associations cross‐sectionally at baseline and longitudinally as part of the SOL‐Investigation of Neurocognitive Aging (SOL‐INCA) ancillary study. Additionally, we examined these associations overall, by sex (males vs. females), and by sex‐specific cancer sites (cervix, female breast, uterus, and prostate). Given that cancers of the cervix and uterus are diagnosed at later stages in Hispanics/Latinas, and are more aggressive relative to cancers of the breast or prostate,^18^ we hypothesized that a history of cervical or endometrial cancers would be associated with poorer cognitive test performance and with greater declines in cognitive test performance over time.

## 2 METHODS

### 2.1 Study design

The HCHS/SOL is a multicenter, community‐based, prospective cohort study designed to identify risk and protective factors influencing the health US Hispanics/Latinos.^19^ Hispanic/Latino adults, ages 18–74, were identified using a stratified two‐stage area probability sampling, with stratification and oversampling incorporated at each stage across four US metropolitan areas–Miami, FL; San Diego, CA; Chicago, IL; and the Bronx area of NY, as previously described.^19^, ^20^ The complex survey sampling procedures used in HCHS/SOL were designed to yield representative data for Hispanics/Latinos in each target city. At baseline in 2008–2011 (Visit 1, V1), the 16,415 Hispanic/Latino adults who enrolled into HCHS/SOL underwent an extensive clinical exam and completed an interviewer‐administered questionnaire that elicited information on risk and protective factors for chronic health conditions. HCHS/SOL participants 45 or older (*n* = 9714) were also administered a neurocognitive battery by trained bilingual/bicultural technicians, which was repeated at a 7‐year follow‐up visit (Visit 2, V2) by those who returned (*n* = 6377) in 2015–2018 as part of the Study of Latinos–Investigation of Neurocognitive Aging (SOL‐INCA) ancillary study.^21^ SOL‐INCA uses the complex design features of HCHS/SOL and probability weights that account for non‐response and attrition.^21^ In the present study, we excluded 75 HCHS/SOL participants who were missing self‐reported history of cancer at V1 resulting in analytic samples of 9639 in cross‐sectional analyses and 6377 in longitudinal analyses. The mean age of the target population at V1 was 56.39 (standard error, SE = 0.14), 54.7% were female, the majority self‐reported their heritage as Mexican (30.8%), Cuban (27.2%), or Puerto Rican (18.1%), 90.8% were foreign‐born, and 38.7% had greater high school education.

The HCHS/SOL and the SOL‐INCA studies were reviewed and approved by the Institutional Review Boards of San Diego State University and the University of California, San Diego, and all participating sites. All participants provided written informed consent prior to participating in study activities.

### 2.2 Neurocognitive assessments

The tests included in the neurocognitive battery and their development, validation, and administration procedures have been previously described in detail.^21^, ^22^ The neurocognitive battery included the Six‐Item Screener (SIS) ^23^; the Brief‐Spanish English Verbal Learning Test (B‐SEVLT) ^24^, ^25^; the Word Fluency Test (WF) ^26^; and the Digit Symbol Substitution Test (DSS).^27^ The SIS is a screening measure for cognitive impairment that consists of six items from the Mini‐Mental State Examination (MMSE) ^28^ including three recall items and three temporal orientation items.^23^ The SIS was scored as the total number of correct items (scores range from 0 to 6); a score of ≥3 has high sensitivity (88.7%) and specificity (88.0%) for identifying cognitive impairment.^23^ The B‐SEVLT is a measure of episodic learning and verbal memory that consists of a list of 15 items that are presented at a pace of 1.5 seconds per word with an immediate recall trial after each complete list presentation.^24^ After three learning trials, an interference procedure is introduced in which participants are asked to repeat aloud words from a separate 15‐word list. Immediately following the interference procedure, delayed free‐recall is tested for the first list. The B‐SEVLT was scored as the total number of items correctly recalled across each of the three learning trials (B‐SEVLT‐Sum, scores range from 0 to 45), and the total number of items correctly recalled during the delayed recall trial (B‐SEVLT‐Recall, scores range from 0 to 15). The WF is a measure of language and executive functioning in which participants are given 60 seconds to produce as many unique words as possible starting with the letter “F” and 60 seconds to produce as many unique words as possible starting with the letter “A”.^26^ Participants were allowed to produce words in either English or Spanish, but were asked to leave out names of people or places and numbers. The WF was scored was scored as the total number of unique correct words produced in both trials (scores range from 0 to 49). The DSS is a measure of psychomotor speed that requires participants to match symbols to numbers according to a key located on the top of the page and to copy the symbols into spaces below the row of numbers.^27^ The DSS was scored as the number of correct symbols completed in 90 seconds (scores range from 0 to 83).

Participants completed all measures in their preferred language in either Spanish or English.

### 2.3 Cancer history

As part of the V1 questionnaire, participants were asked to report on their medical history including cancer. Participants were asked: “Has a doctor ever said that you have cancer or a malignant tumor?” Participants who reported in the affirmative were asked to report on the type(s) of cancer(s) they were diagnosed from a list of 14 cancers including cancers of the lung, breast, cervix, blood/lymph glands, testes/scrotum, bone, skin (melanoma or non‐melanoma), brain, stomach, colon, uterus, prostate, or liver. Participants were also provided with an “other” response category, for cancers not listed. Prior cohort studies have demonstrated a high degree of accuracy for this self‐reported method.^29^

### 2.4 Covariates

Potential confounders were selected based on previous epidemiologic studies of Alzheimer's disease and dementia^30^ and using directed acyclic graphs.^31^ Covariates included: age (continuous in years), sex (male or female), education (<high school, high school graduate, or > high school), Hispanic/Latino heritage (Mexican, Puerto Rican, Cuban, Dominican, Central American, South American, or More than one/Other), and nativity (born in 50 US states, yes or no). Given that the timeframe for the reporting of cancers was prior to baseline, behaviors assessed at baseline including cigarette smoking, diet, and physical activity, and health conditions including obesity, hypertension, diabetes, and anxiety were identified as potential mediators (i.e., they are influenced by a diagnosis of cancer and increase the risk of cognitive decline), and thus were not included in the adjustment set.

### 2.5 Statistical analysis

All cognitive outcomes were standardized to facilitate the comparison of estimated associations across tests as follows. We calculated cognitive change scores using regression‐based methods^32^; we used survey‐weighted linear regression models where we regressed cognitive performance at V2 as a function of cognitive performance at V1 and elapsed days between assessments. Test‐specific standardized measures of change were subsequently calculated as (V2‐V2_pred_)/RMSE, where V2 is the respondent's cognitive score at V2, V2_pred_ is their predicted score at V2, and RMSE is the regression‐derived root mean squared error (RMSE). We generated a global cognitive change measure by averaging the standardized scores of B‐SEVLT‐SUM, B‐SEVLT‐RECALL, WF, and DSS.^21^

We examined descriptive characteristics of the study population at V1 using survey weighted means with SEs and *n*'s with survey‐weighted percentages. We used survey linear regression to examine the cross‐sectional associations [β estimates, SEs, and *p*‐values] between a history of cancer and neurocognitive test performance at V1, and the longitudinal associations between a history of cancer and differences in neurocognitive test performance from V1 to V2 among those without cognitive impairment at V1 (SIS ≥ 3). We adjusted base models for age, sex, and education, and fully‐adjusted models for age, sex, education, Hispanic/Latino heritage, and nativity. We examined associations among all participants overall, by sex (females vs. males), and by the most frequently reported cancer sites including cervix, female breast, uterus, and prostate. In sensitivity analyses, we excluded non‐melanoma skin cancer from our definition of cancer history given the high survival rate.^33^ All analyses were performed using R Version 4.2 (R Foundation for Statistical Computing) and were based on participants with complete data (i.e., complete‐case analysis). All statistical tests were two‐sided, and a *p* value of less than 0.05 was considered statistically significant.

## 3 RESULTS

The characteristics for the target population overall and by self‐reported history of cancer are shown in Table 1. Compared to those without a history of cancer, at baseline those with cancer history were older (60.50 [SE = 0.54] versus 56.10 [SE = 0.14] years), and higher proportions of those with cancer history were female (65.0% vs. 54.0%), had <high school education (43.8% vs. 39.7%), were former smokers (29.8% vs. 25.3%), and reported engaging in high levels of physical activity (54.1% vs. 49.4%). Among those who reported a history of cancer (unweighted *n* = 546), the cancer sites reported included (in descending frequency and not mutually exclusive; unweighted *n*, weighted %): cervix (121, 18.1%), female breast (115, 20.2%), uterus (67, 12.8%), prostate (48, 11.5%), skin [non‐melanoma] (42, 11.6%), colon (26, 5.2%), blood/lymph glands (20, 2.7%), stomach (15, 2.1%), skin [melanoma] (13, 2.5%), liver (10, 1.4%), lung (9, 1.9%), brain (9, 1.8%), bone (4, 0.6%), and testes/scrotum (1, 0.4%). A cancer other than those listed was reported by 113 individuals (22.0% of the target population).

**TABLE 1:** HCHS/SOL target population baseline characteristics (2008–2011), overall and by self‐reported history of cancer.

Characteristic	Overall	Baseline history of cancer	
		No	Yes
	%	%	%
Unweighted *n*	9639	9093	546
Weighted %	100.0	93.6	6.4

Age in years, mean [SE]	56.39 [0.14]	56.10 [0.14]	60.50 [0.54]
Sex			
Female	54.7	54.0	65.0
Male	45.3	46.0	35.0
Hispanic/Latino heritage			
Mexican	30.8	31.5	20.8
Cuban	27.2	26.4	38.6
Puerto Rican	18.1	17.9	22.0
Dominican	9.4	9.6	6.7
Central American	6.7	6.7	5.6
South American	5.5	5.6	4.3
More than One/Other	2.3	2.3	2.0
US born (50 US states)			
No	90.8	90.8	90.8
Yes	9.2	9.2	9.2
Language preference			
Spanish	85.8	85.6	89.2
English	14.2	14.4	10.8
Education			
<High school	40.0	39.7	43.8
High school graduate	21.3	21.7	15.6
>High school	38.7	38.6	40.6
BMI^a^ in kg/m^2^, mean [SE]	29.87 [0.09]	29.84 [0.09]	30.27 [0.34]
Smoking status			
Never smoker	53.8	53.7	55.7
Former smoker	25.6	25.3	29.8
Current smoker	20.6	21.0	14.5
AHEI‐2010^b^	50.20 [0.20]	50.27 [0.21]	49.07 [0.38]
Physical activity^c^			
Low MVPA	8.1	8.3	3.9
Moderate MVPA	42.3	42.3	42.0
High MVPA	49.7	49.4	54.1
Diabetes^d^			
No diabetes	24.8	25.3	18.6
Pre‐diabetes	45.2	45.7	37.6
Treated diabetes	17.3	16.6	26.9
Untreated diabetes	12.7	12.5	16.9
Hypertension^e^			
No	56.3	57.3	40.9
Yes	43.7	42.7	59.1
Depression^f^			
CESD10 score < 10	68.7	69.2	62.6
CEST10 score ≥ 10	31.3	30.8	37.4
Anxiety^g^			
STAI10 score ≤ 16	55.1	55.4	52.2
STAI10 score > 16	44.9	44.6	47.8

Hispanic Community Health Study/Study of Latinos (HCHS/SOL) participants completed all baseline assessments in 2008–2011.

Abbreviations: AHEI‐2010, Alternative Healthy Eating Index; BMI, body mass index; SE, standard error; US, United States.

^a^ Derived from measured height and weight; continuous in kg/m^2^.

^b^ Assessed using the alternative healthy eating index (AHEI‐2010).

^c^ Assessed using the Global Physical Activity Questionnaire (GPAQ).

^d^ Defined using serum glucose levels adjusted for fasting time and, if available, post‐oral glucose tolerance test glucose levels, hemoglobin A1c, self‐reported diabetes, and use of anti‐diabetes medications.

^e^ Measured systolic or diastolic blood pressure > =140/90 or current use of antihypertensive medications.

^f^ Assessed using the 10‐item Center for Epidemiologic Studies Depression Scale.

^g^ Assessed using the 10‐item State–Trait Anxiety Inventory; dichotomized at the median.

The cross‐sectional and longitudinal associations between a history of cancer and neurocognitive test performance are reported in Table 2. At V1, a history of cancer (vs. no history of cancer) was associated with better performance on the WF among the entire population (β = 0.14, SE = 0.06; *p* = 0.03); and with higher global cognition among the entire population (β = 0.09, SE = 0.04; *p* = 0.04) and among men (β = 0.11, SE = 0.06; *p* = 0.06). These associations were robust to the exclusion of non‐melanoma skin cancers (Table S1). From V1 to V2, a history of cancer was associated with increases in performance on the B‐SEVLT‐Sum (β = 0.33, SE = 0.15; *p* = 0.03) and on the B‐SEVLT‐Recall (β = 0.38, SE = 0.16; *p* = 0.02) among men, and with non‐statistically significant decreases in performance on the B‐SEVLT‐Sum (β = −0.12, SE = 0.11; *P* = 0.30) and on the B‐SEVLT‐Recall (β = −0.11, SE = 0.09; *p* = 0.25) among women. Among men, the association was attenuated and not statistically significant for B‐SEVLT‐Sum (β = 0.30, SE = 0.16; *p* = 0.06) and of larger magnitude and statistically significant for B‐SEVLT‐Recall (β = 0.43, SE = 0.17; *p* = 0.01) when we excluded non‐melanoma skin cancers (Table S1).

**TABLE 2:** Associations of cross‐sectional and longitudinal neurocognitive test performance and history of cancer, overall and by sex.

	Overall		Females		Males		*P*_Interaction_ ^a^
	β (SE)	*p*	β (SE)	*p*	β (SE)	*p*	
Unweighted *n*	546 cancer/		412 cancer/		134 cancer/		
	9093 no cancer		5581 no cancer		3512 no cancer		
B‐SEVLT‐Sum							
Cross‐sectional (V1)							
Model 1^b^	0.03 (0.06)	0.58	0.02 (0.08)	0.82	0.06 (0.08)	0.43	0.65
Model 2^c^	0.07 (0.06)	0.23	0.05 (0.07)	0.48	0.10 (0.08)	0.24	0.49
Longitudinal (V1–V2)							
Model 1^b^	0.03 (0.09)	0.73	−0.10 (0.11)	0.36	0.33 (0.16)	0.04	0.03
Model 2^c^	0.02 (0.09)	0.80	−0.12 (0.11)	0.30	0.33 (0.15)	0.03	0.02
B‐SEVLT‐Recall Cross‐sectional (V1)							
Model 1^b^	0.02 (0.06)	0.69	0.00 (0.07)	0.99	0.07 (0.09)	0.46	0.50
Model 2^c^	0.06 (0.05)	0.27	0.03 (0.07)	0.65	0.11 (0.09)	0.22	0.36
Longitudinal (V1–V2)							
Model 1^b^	0.05 (0.09)	0.56	−0.10 (0.09)	0.27	0.39 (0.17)	0.02	0.01
Model 2^c^	0.05 (0.08)	0.55	−0.11 (0.09)	0.25	0.38 (0.16)	0.02	0.01
WF Cross‐sectional (V1)							
Model 1^b^	0.10 (0.06)	0.13	0.11 (0.08)	0.18	0.06 (0.09)	0.47	0.86
Model 2^c^	0.14 (0.06)	0.03	0.14 (0.08)	0.09	0.11 (0.08)	0.18	0.77
Longitudinal (V1–V2)							
Model 1^b^	0.12 (0.10)	0.23	0.09 (0.11)	0.45	0.21 (0.20)	0.30	0.77
Model 2^c^	0.12 (0.10)	0.26	0.08 (0.11)	0.50	0.21 (0.20)	0.30	0.80
DSS Cross‐sectional (V1)							
Model 1^b^	0.06 (0.05)	0.23	0.04 (0.06)	0.49	0.10 (0.09)	0.26	0.31
Model 2^c^	0.08 (0.05)	0.14	0.03 (0.06)	0.55	0.14 (0.09)	0.10	0.10
Longitudinal (V1–V2)							
Model 1^b^	−0.07 (0.08)	0.32	−0.13 (0.08)	0.11	0.07 (0.19)	0.72	0.35
Model 2^c^	−0.06 (0.08)	0.41	−0.12 (0.08)	0.13	0.06 (0.19)	0.74	0.36
Global Cognition Cross‐sectional (V1)							
Model 1^b^	0.06 (0.04)	0.20	0.04 (0.05)	0.41	0.07 (0.06)	0.24	0.56
Model 2^c^	0.09 (0.04)	0.04	0.07 (0.05)	0.22	0.11 (0.06)	0.06	0.27
Longitudinal (V1–V2)							
Model 1^b^	0.03 (0.06)	0.59	−0.06 (0.05)	0.22	0.24 (0.15)	0.11	0.06
Model 2^c^	0.03 (0.06)	0.62	−0.07 (0.05)	0.18	0.24 (0.15)	0.10	0.05

Hispanic Community Health Study/Study of Latinos (HCHS/SOL) participants completed all baseline assessments in 2008–2011. HCHS/SOL participants completed follow‐up assessments as part of the Study of Latinos‐Investigation of Neurocognitive Aging (SOL‐INCA) ancillary study in 2015–2018.

B‐SEVLT, brief‐spanish english verbal learning test; DSS, digit symbol substitution test; WF, word fluency test.

^a^ *p* Interaction is the *p‐*value for the multiplicative interaction between baseline history of cancer and sex.

^b^ Model 1 is adjusted for age, sex, and education.

^c^ Model 2 is adjusted for age, sex, education, Hispanic/Latino heritage, nativity, and language preference.

The cross‐sectional and longitudinal associations between a history of cancer and neurocognitive test performance by the most frequently reported cancer sites are reported in Table 3. At V1, among men, a history of prostate cancer was associated with better performance on the WF (β = 0.29, SE = 0.12; *p* = 0.02) and among women, a history of cervical cancer was associated with declines in performance on the B‐SEVLT‐Recall (β = −0.31, SE = 0.13; *p* = 0.02). We did not observe any associations between a history of cancer and cognitive performance cross‐sectionally or longitudinally for female breast or uterine cancers, although for breast cancer, all of the longitudinal associations except for WF were in the direction of declines in neurocognitive performance.

**TABLE 3:** Associations of cross‐sectional and longitudinal neurocognitive test performance and history of cancer by sex‐specific cancer sites.

	Cervical cancer		Female breast cancer		Uterine cancer		Prostate cancer	
	β (SE)	*p*	β (SE)	*p*	β (SE)	*p*	β (SE)	*p*
Unweighted *n*	121 cancer/		115 cancer/		67 cancer/		48 cancer/	
	5581 no cancer		5581 no cancer		5581 no cancer		5581 no cancer	
B‐SEVLT‐Sum								
Cross‐sectional (V1)								
Model 1^a^	−0.02 (0.14)	0.90	−0.01 (0.15)	0.96	−0.15 (0.31)	0.63	0.01 (0.13)	0.93
Model 2^b^	0.01 (0.13)	0.94	−0.01 (0.14)	0.95	−0.03 (0.28)	0.92	0.02 (0.14)	0.90
Longitudinal (V1–V2)								
Model 1^a^	−0.14 (0.14)	0.32	−0.29 (0.24)	0.23	0.05 (0.17)	0.78	0.44 (0.24)	0.07
Model 2^b^	−0.13 (0.16)	0.39	−0.31 (0.25)	0.22	0.03 (0.14)	0.80	0.46 (0.22)	0.04
B‐SEVLT‐Recall Cross‐sectional (V1)								
Model 1^a^	0.02 (0.09)	0.87	−0.03 (0.13)	0.84	0.03 (0.19)	0.85	0.23 (0.14)	0.09
Model 2^b^	0.04 (0.10)	0.72	−0.01 (0.12)	0.91	0.16 (0.17)	0.35	0.25 (0.14)	0.06
Longitudinal (V1–V2)								
Model 1^a^	−0.33 (0.14)	0.02	−0.11 (0.17)	0.54	0.02 (0.24)	0.93	0.35 (0.24)	0.14
Model 2^b^	−0.31 (0.13)	0.02	−0.11 (0.17)	0.51	0.04 (0.21)	0.86	0.37 (0.23)	0.11
WF Cross‐sectional (V1)								
Model 1^a^	0.16 (0.19)	0.42	−0.01 (0.12)	0.96	−0.13 (0.17)	0.44	0.27 (0.12)	0.03
Model 2^b^	0.18 (0.20)	0.35	0.00 (0.13)	0.98	−0.05 (0.15)	0.75	0.29 (0.12)	0.02
Longitudinal (V1–V2)								
Model 1^a^	0.18 (0.12)	0.13	0.25 (0.25)	0.32	0.02 (0.18)	0.93	0.13 (0.28)	0.64
Model 2^b^	0.18 (0.12)	0.13	0.24 (0.25)	0.35	0.00 (0.19)	0.98	0.12 (0.26)	0.64
DSS Cross‐sectional (V1)								
Model 1^a^	−0.08 (0.09)	0.37	0.13 (0.13)	0.33	−0.03 (0.12)	0.80	0.07 (0.13)	0.56
Model 2^b^	−0.05 (0.08)	0.57	0.14 (0.14)	0.29	−0.02 (0.12)	0.90	0.14 (0.13)	0.27
Longitudinal (V1–V2)								
Model 1^a^	−0.06 (0.12)	0.64	−0.20 (0.13)	0.13	−0.27 (0.17)	0.12	0.06 (0.25)	0.80
Model 2^b^	−0.04 (0.13)	0.78	−0.20 (0.14)	0.15	−0.25 (0.17)	0.14	0.09 (0.26)	0.73
Global Cognition Cross‐sectional (V1)								
Model 1^a^	0.03 (0.10)	0.77	0.03 (0.10)	0.80	−0.06 (0.18)	0.73	0.13 (0.11)	0.23
Model 2^b^	0.05 (0.11)	0.61	0.03 (0.10)	0.72	0.03 (0.16)	0.88	0.16 (0.11)	0.13
Longitudinal (V1–V2)								
Model 1^a^	−0.09 (0.07)	0.19	−0.09 (0.09)	0.33	−0.04 (0.11)	0.71	0.23 (0.18)	0.19
Model 2^b^	−0.08 (0.07)	0.31	−0.10 (0.09)	0.29	−0.04 (0.10)	0.68	0.24 (0.17)	0.16

Hispanic Community Health Study/Study of Latinos (HCHS/SOL) participants completed all baseline assessments in 2008–2011 (Visit 1, V1). HCHS/SOL participants completed follow‐up assessments as part of the Study of Latinos‐Investigation of Neurocognitive Aging (SOL‐INCA) ancillary study in 2015–2018 (Visit 2, V2).

B‐SEVLT, brief‐spanish english verbal learning test; DSS, digit symbol substitution Test; WF, word fluency test.

^a^ Model 1 is adjusted for age and education.

^b^ Model 2 is adjusted for age, education, Hispanic/Latino heritage, nativity, and language preference.

## 4 DISCUSSION

This study examined the associations between a history of cancer and neurocognitive test performance cross‐sectionally and longitudinally over a mean 7‐year follow‐up among middle‐aged and older Hispanic/Latino adults from four US metropolitan areas. Most notably, in this study a history of cervical cancer was associated with 7‐year declines in memory among cognitively healthy, diverse, and middle‐aged Hispanic/Latinas. While not statistically significant, results also showed declines in episodic learning and verbal memory among all women, and with declines in neurocognitive tests performance across most tests among women with breast cancer. Among men, however, a history of cancer was associated with *better* performance on tests of language and executive functioning and episodic learning and verbal memory and with improved overall global cognition over time. To our knowledge, this is the first study to examine the association between a history of cancer and neurocognitive test performance among Hispanic/Latino adults.

Studies of cognitive function among Hispanics/Latinos are limited,^14^ but are needed given that Hispanics/Latinos are often diagnosed with cancer at younger ages than other racial/ethnic groups,^16^ which may alter the trajectory of cognitive decline over a longer period of time, and Hispanics/Latinos are diagnosed with cancer at more advanced stage,^17^ which may require more aggressive, systemic, and potentially neurotoxic therapies resulting in worse cognitive impairment. One recent study of 54 Hispanic/Latina breast cancer survivors examined associations of body composition, diet, and physical activity with cognitive function,^14^ but was limited by a cross‐sectional design, and was not aimed at examining the effects of cancer on cognition.^14^ As summarized in a number of systematic reviews and meta‐analyses of studies of CRCI, most of which have focused on non‐Hispanics/Latinos diagnosed with breast^34^, ^35^ or prostate^36^, ^37^ cancers, studies have consistently reported greater cognitive dysfunction and greater declines in cognitive function among patients treated with systemic therapies as compared to normative samples, study control groups, or pre‐treatment baseline assessments of the same cancer patients. Studies examining CRCI among women with cervical cancer or cervical cancer survivors are limited, but our findings are consistent with prior studies including a cross‐sectional study by Areklett and colleagues^38^ of 254 cervical cancer survivors in Norway who were treated with combined chemo‐radiotherapy or surgery only, and a longitudinal study by Kirchheiner and colleagues^39^ of 744 EMBRACE Study participants with locally advanced cervical cancer who underwent definitive chemoradiation therapy with image guided adaptive brachytherapy. In the study by Areklett and colleagues, 42.5% of women reported significant cognitive impairment as assessed by self‐report using the Functional Assessment of Cancer Therapy‐Cognitive Function (FACT‐Cog), and receipt of combined chemo‐radiotherapy was associated with a four‐fold increase in the odds of CRCI.^38^ In the study by Kirchheiner and colleagues, cognitive function was assessed using the European Organization for Research and Treatment of Cancer (EORTC) Quality of Life Questionnaire C30 (QLQ‐C30) at baseline (before treatment), and every 3 months after treatment during the first year, then every 6 months during the second and third years, and yearly thereafter. In their study, concentration and memory were stable over time but impaired to a clinically relevant degree as compared to a Dutch female general reference population. Virtually all classes of chemotherapeutics and biologics including mitotic inhibitors, antimetabolites, and DNA cross‐linking agents have been shown to result in adverse neurological effects with animal studies demonstrating increases in apoptosis, changes in cell morphology, impairments in neurogenesis, and increases neuroinflammation.^40^ Taken together, findings from these human and animal studies suggest strong adverse effects on cognition and brain health among women as a result of more aggressive and systemic therapies.

In contrast with most,^2^ but not all of the published literature,^41^ in this study we found that Hispanic/Latino older adults with a history of cancer and in particular men, and men with a history of prostate cancer, performed better on tests of verbal memory and showed improvements in performance on tests of episodic learning, verbal memory, and language over time as compared with adults without a history of cancer. Similar findings were reported in the population‐based Health and Retirement Study; adults who developed cancer had better memory and slower memory decline than did cancer‐free individuals.^41^ While these findings are in line with prior observations that changes in cognitive function are reversible for many but not all cancer survivors naturally^42^, ^43^ or through cognitive rehabilitation,^44^, ^45^, ^46^, ^47^ or observations that cancer patients and survivors may have compensatory and more effortful processing,^48^ another plausible explanation is that cancer survivors may have altered their behaviors in order to improve prognosis or quality of life after cancer,^49^ and these changes in behaviors may have also led to subsequent improvements in cognition over time. This hypothesis is supported by our observations that those with a history of cancer in our study were more likely to be former smokers and reported engaging in higher levels of physical activity compared to those without a history of cancer.

This study had a number of strengths including the assessment of cognitive battery of the neuropsychological tests with robust normative data for diverse Hispanics/Latinos. Second, longitudinal 7‐year cognitive assessments of middle‐aged and older Hispanic/Latino adults in relation to self‐reported cancer histories in an understudied population, provide unique opportunities to understand relationships between cancer, sex, and cognitive aging that may extend to other racial or ethnic groups. Limitations include the fact that we relied on self‐reported cancer history and did not have details about dates of cancer diagnosis, tumor characteristics, and cancer treatments, limiting our ability to comment on the specific aspects of cancer that may be causally related to cognitive function and decline in this population. As such, we were unable to adjust the cross‐sectional analyses for time since cancer diagnosis, which is likely to vary greatly. Large studies of Hispanics/Latinos will be required to fully understand cognition within the context of specific cancers and cancer treatments in this population. Importantly, research on cancer and CRCI has progressed from a pharmacotoxicology perspective to a more holistic view of cognitive changes where treatment, psychological effects, and individual characteristics, factors, or behaviors that increase susceptibility to cognitive decline following cancer interact.^2^ Thus, studies that consider the complex pathways by which cancer may impact cognition are also needed. We also relied on neurocognitive assessments that were not developed specifically to assess cognitive impairment directly resulting from cancer or cancer treatments, but rather are used to examine the cognitive consequences of brain aging, damage, and disease.^50^ More subtle changes in CRCI may be missed by these assessments; however, these tests allow us to identify specific domains of cognitive function and abilities, and when combined with additional testing modalities such as brain imaging, may provide powerful insights into the effects of cancer on cognition. In this study, we observed low to medium effect sizes; however, extrapolating to the population‐level, these findings can yield significant public health and associated community level health improvements if addressed. As in other studies of older adults and in particular adults with cancer, our study may be subject to selection bias due to selective mortality.^51^ Last, some associations may be spurious due to the large number of statistical tests conducted; however, as cancer and its treatments are known to exert physiologic and psychological effects, real associations are expected. As such, adjustments for multiple comparisons are unwarranted.^52^

## 5 CONCLUSIONS

Among diverse middle‐aged and older Hispanic/Latinos, self‐reported cervical cancer was associated with 7‐year cognitive decline among Hispanic/Latina women, and self‐reported history of cancer and in particular prostate cancer was associated with improvements in cognition over time among men. The impact of cancer on cognitive aging and impairment remains to be elucidated as this cohort ages, which is increasingly important as the number of Hispanic/Latino cancer survivors continues to grow.

## AUTHOR CONTRIBUTIONS

**Margaret S. Pichardo:** Writing – review and editing (supporting). **Linda C. Gallo:** Data curation (equal); funding acquisition (equal); project administration (equal); resources (equal); writing – review and editing (equal). **Gregory A. Talavera:** Data curation (equal); funding acquisition (equal); resources (equal); writing – review and editing (equal). **Corinne McDaniels‐Davidson:** Writing – review and editing (supporting). **Frank J Penedo:** Writing – review and editing (supporting). **David J Lee:** Writing – review and editing (supporting). **Wassim Tarraf:** Writing – review and editing (supporting). **Tanya P. Garcia:** Writing – review and editing (supporting). **Martha L. Daviglus:** Writing – review and editing (supporting). **Hector M. Gonzalez:** Data curation (equal); funding acquisition (equal); resources (equal); writing – review and editing (supporting).

## FUNDING INFORMATION

This work is supported by the National Institute on Aging (NIA), National Institutes of Health (NIH) (R01 AG048642, R56 AG048642 and R01 AG075758). H Parada Jr was supported by the National Cancer Institute (K01 CA234317), the SDSU/UCSD Comprehensive Cancer Center Partnership (U54 CA132384 and U54 CA132379), and the Alzheimer's Disease Resource Center for advancing Minority Aging Research at the University of California San Diego (P30 AG059299). H.M.G. also receives support from NIH/NIA P30 AG062429 and P30 AG059299. The HCHS/SOL was carried out as a collaborative study supported by contracts from the National Heart, Lung, and Blood Institute (NHLBI) to the University of North Carolina (N01‐HC65233), University of Miami (N01‐HC65234), Albert Einstein College of Medicine (N01‐HC65235), Northwestern University (N01‐HC65236), and San Diego State University (N01‐HC65237). The following institutes/centers/offices contribute to the HCHS/SOL through a transfer of funds to the NHLBI: National Institute on Minority Health and Health Disparities, National Institute on Deafness and Other Communication Disorders, National Institute of Dental and Craniofacial Research, National Institute of Diabetes and Digestive and Kidney Diseases, National Institute of Neurological Disorders and Stroke, and Office of Dietary Supplements, NIH. The contents of this work are solely the responsibility of the authors and do not necessarily represent the official views of the NIH. The NIH and NIA role was limited to providing financial research support.

## CONFLICT OF INTEREST STATEMENT

The authors declare they have no conflict of interest.

## Supporting information


Table S1.

## Data Availability

The data that support the findings of this study are available on request from the Hispanic Community Health Study/Study of Latinos: https://sites.cscc.unc.edu/hchs/. The data are not publicly available due to privacy or ethical restrictions.
