# Supplementary material for: Neurocognitive test performance following cancer among middle‐aged and older adults in the Hispanic Community Health Study/Study of Latinos (HCHS/SOL) and the SOL‐Investigation of Neurocognitive Aging Ancillary Study
Source: Cancer Med. 2023 Mar 31;12(10):11860–70. doi: 10.1002/cam4.5863 (PMC10242865; doi:10.1002/cam4.5863)
Supplement: Supplementary file 1 — Table S1. [file CAM4-12-11860-s001.docx]

**Supplemental eTable 1.** Associations of cross-sectional and longitudinal neurocognitive test performance and history of cancer excluding non-melanoma skin cancer.

|  | **Overall** | |  | **Females** | |  | **Males** | |  | ***P*_Interaction_** |
| --- | --- | --- | --- | --- | --- | --- | --- | --- | --- | --- |
|  | **β (SE)** | ***P*** |  | **β (SE)** | ***P*** |  | **β (SE)** | ***P*** |  |  |
| *Unweighted n* | 504 cancer/ | |  | 386 cancer/ | |  | 118 cancer/ | |  |  |
|  | 9,093 no cancer | |  | 5,581 no cancer | |  | 3,512 no cancer | |  |  |
|  |  |  |  |  |  |  |  |  |  |  |
| **B-SEVLT-Sum** |  |  |  |  |  |  |  |  |  |  |
| Cross-sectional (V1) |  |  |  |  |  |  |  |  |  |  |
| Model 1^a^: | 0.03 (0.06) | 0.61 |  | -0.01 (0.08) | 0.89 |  | 0.13 (0.09) | 0.16 |  | 0.21 |
| Model 2^b^: | 0.06 (0.06) | 0.30 |  | 0.02 (0.07) | 0.80 |  | 0.15 (0.09) | 0.10 |  | 0.15 |
| Longitudinal (V1to V2) |  |  |  |  |  |  |  |  |  |  |
| Model 1^a^: | 0.02 (0.11) | 0.86 |  | -0.10 (0.13) | 0.42 |  | 0.30 (0.18) | 0.09 |  | 0.06 |
| Model 2^b^: | 0.01 (0.11) | 0.89 |  | -0.12 (0.13) | 0.37 |  | 0.30 (0.16) | 0.06 |  | 0.04 |
|  |  |  |  |  |  |  |  |  |  |  |
| **B-SEVLT-Recall** |  |  |  |  |  |  |  |  |  |  |
| Cross-sectional (V1) |  |  |  |  |  |  |  |  |  |  |
| Model 1^a^: | 0.02 (0.06) | 0.69 |  | -0.03 (0.07) | 0.73 |  | 0.12 (0.09) | 0.18 |  | 0.16 |
| Model 2^b^: | 0.06 (0.06) | 0.32 |  | 0.00 (0.07) | 0.96 |  | 0.16 (0.10) | 0.09 |  | 0.11 |
| Longitudinal (V1to V2) |  |  |  |  |  |  |  |  |  |  |
| Model 1^a^: | 0.02 (0.10) | 0.84 |  | -0.16 (0.11) | 0.13 |  | 0.43 (0.18) | 0.02 |  | <0.01 |
| Model 2^b^: | 0.02 (0.10) | 0.83 |  | -0.16 (0.10) | 0.12 |  | 0.43 (0.17) | 0.01 |  | <0.01 |
|  |  |  |  |  |  |  |  |  |  |  |
| **WF** |  |  |  |  |  |  |  |  |  |  |
| Cross-sectional (V1) |  |  |  |  |  |  |  |  |  |  |
| Model 1^a^: | 0.10 (0.07) | 0.14 |  | 0.11 (0.09) | 0.23 |  | 0.07 (0.09) | 0.40 |  | 0.96 |
| Model 2^b^: | 0.13 (0.07) | 0.05 |  | 0.13 (0.09) | 0.14 |  | 0.12 (0.08) | 0.16 |  | 0.72 |
| Longitudinal (V1to V2) |  |  |  |  |  |  |  |  |  |  |
| Model 1^a^: | 0.14 (0.11) | 0.19 |  | 0.12 (0.12) | 0.33 |  | 0.20 (0.22) | 0.36 |  | 0.92 |
| Model 2^b^: | 0.14 (0.11) | 0.21 |  | 0.12 (0.12) | 0.35 |  | 0.20 (0.22) | 0.37 |  | 0.95 |
|  |  |  |  |  |  |  |  |  |  |  |
| **DSS** |  |  |  |  |  |  |  |  |  |  |
| Cross-sectional (V1) |  |  |  |  |  |  |  |  |  |  |
| Model 1^a^: | 0.08 (0.05) | 0.15 |  | 0.05 (0.06) | 0.38 |  | 0.11 (0.09) | 0.20 |  | 0.33 |
| Model 2^b^: | 0.09 (0.05) | 0.08 |  | 0.05 (0.06) | 0.38 |  | 0.15 (0.09) | 0.09 |  | 0.15 |
| Longitudinal (V1to V2) |  |  |  |  |  |  |  |  |  |  |
| Model 1^a^: | -0.06 (0.09) | 0.49 |  | -0.15 (0.08) | 0.08 |  | 0.16 (0.23) | 0.49 |  | 0.22 |
| Model 2^b^: | -0.06 (0.09) | 0.51 |  | -0.14 (0.09) | 0.10 |  | 0.15 (0.23) | 0.51 |  | 0.23 |
|  |  |  |  |  |  |  |  |  |  |  |
| **Global Cognition** |  |  |  |  |  |  |  |  |  |  |
| Cross-sectional (V1) |  |  |  |  |  |  |  |  |  |  |
| Model 1^a^: | 0.06 (0.05) | 0.20 |  | 0.03 (0.06) | 0.55 |  | 0.11 (0.06) | 0.09 |  | 0.25 |
| Model 2^b^: | 0.09 (0.04) | 0.05 |  | 0.05 (0.06) | 0.33 |  | 0.14 (0.06) | 0.02 |  | 0.12 |
| Longitudinal (V1to V2) |  |  |  |  |  |  |  |  |  |  |
| Model 1^a^: | 0.03 (0.07) | 0.68 |  | -0.07 (0.06) | 0.19 |  | 0.26 (0.17) | 0.11 |  | 0.06 |
| Model 2^b^: | 0.03 (0.07) | 0.68 |  | -0.08 (0.06) | 0.18 |  | 0.26 (0.16) | 0.10 |  | 0.05 |
| Hispanic Community Health Study/Study of Latinos (HCHS/SOL) participants completed all baseline assessments in 2008-2011 (Visit 1, V1). HCHS/SOL participants completed follow-up assessments as part of the Study of Latinos-Investigation of Neurocognitive Aging (SOL-INCA) ancillary study in 2015-2018 (Visit 2, V2).  B-SEVLT, Brief-Spanish English Verbal Learning Test; DSS, Digit Symbol Substitution Test; WF, Word Fluency Test  ^a^ Model 1 is adjusted for age, sex, and education.  ^b^ Model 2 is adjusted for age, sex, education, Hispanic/Latino heritage, nativity, and language preference. | | | | | | | | | | |
